# Fucoxanthin-Containing Cream Prevents Epidermal Hyperplasia and UVB-Induced Skin Erythema in Mice

**DOI:** 10.3390/md16100378

**Published:** 2018-10-10

**Authors:** Azahara Rodríguez-Luna, Javier Ávila-Román, María Luisa González-Rodríguez, María José Cózar, Antonio M Rabasco, Virginia Motilva, Elena Talero

**Affiliations:** 1 Department of Pharmacology, Faculty of Pharmacy, Universidad de Sevilla, 41012 Seville, Spain; arodriguez53@us.es (A.R.-L.); javieravila@us.es (J.Á.-R.); motilva@us.es (V.M.); 2 Department of Pharmaceutical Technology, Faculty of Pharmacy, Universidad de Sevilla, 41012 Seville, Spain; malugoro@us.es (M.L.G.-R.); cozar@us.es (M.J.C.); amra@us.es (A.M.R.)

**Keywords:** fucoxanthin, inflammation, epidermal hyperplasia, UVB, photoprotection

## Abstract

Microalgae represent a source of bio-active compounds such as carotenoids with potent anti-inflammatory and antioxidant properties. We aimed to investigate the effects of fucoxanthin (FX) in both in vitro and in vivo skin models. Firstly, its anti-inflammatory activity was evaluated in LPS-stimulated THP-1 macrophages and TNF-α-stimulated HaCaT keratinocytes, and its antioxidant activity in UVB-irradiated HaCaT cells. Next, in vitro and ex vivo permeation studies were developed to determine the most suitable formulation for in vivo FX topical application. Then, we evaluated the effects of a FX-containing cream on TPA-induced epidermal hyperplasia in mice, as well as on UVB-induced acute erythema in hairless mice. Our results confirmed the in vitro reduction of TNF-α, IL-6, ROS and LDH production. Since the permeation results showed that cream was the most favourable vehicle, FX-cream was elaborated. This formulation effectively ameliorated TPA-induced hyperplasia, by reducing skin edema, epidermal thickness, MPO activity and COX-2 expression. Moreover, FX-cream reduced UVB-induced erythema through down-regulation of COX-2 and iNOS as well as up-regulation of HO-1 protein via Nrf-2 pathway. In conclusion, FX, administered in a topical formulation, could be a novel natural adjuvant for preventing exacerbations associated with skin inflammatory pathologies as well as protecting skin against UV radiation.

## 1. Introduction

Skin is the organ that acts as main defence against external environment factors, protecting the organism from harmful substances, mechanical damage, pathological invasion and radiations. Nonetheless, the normal structure and functionality could be altered by external factors such as toxic compounds or ultraviolet (UV) radiation, or by internal factors including genetic predisposition, immune and hormone disorders, or stress. The result of these skin perturbations could trigger an inflammatory process, an oxidative stress status, an unbalanced epidermal homeostasis, or a limited immune response, among others [1].

In this respect, inflammatory skin diseases such as psoriasis, atopic dermatitis or rosacea have harsh clinical implications because of their chronic curse and lead to develop comorbidities that make difficult their treatment and have a remarkable impact on the quality-of-life of patients [2]. Nowadays, UV skin exposure to treat these inflammatory conditions is recommended due to its beneficial effects. In this line, UV radiation achieves long remission periods in psoriasis through activation of immunosuppressive pathways and keratinocyte apoptosis [3]. On the other hand, detrimental effects of UVB radiation exposure (280–315 nm) have been extensively reported: it promotes a strong acute inflammatory response characterized by activation and recruitment of innate immune cells such as neutrophils and macrophages to the epidermis and dermis. In addition, UVB radiation is the main source of reactive oxygen species (ROS) production, which increases the inflammatory response, causing DNA oxidative damage in keratinocytes [4]. For this reason, new approaches to modulate the skin inflammation and protect against UV radiation are necessary to supplement the existing skin anti-inflammatory therapies.

Currently, marine resources are recognized for their variety of biologically active substances [5], which are becoming important ingredients in skin care products due to their potent anti-inflammatory and antioxidant actions as well as the safety and low risk in their administration [6]. In this sense, carotenoids have shown antioxidant, anti-inflammatory or anti-carcinogenic properties in different skin inflammatory models [7]. Fucoxanthin (FX) is an orange carotenoid present in brown seaweeds, diatoms and microalgae [8], whose antioxidant activity has been well demonstrated in previous studies [9,10]. Particularly, FX has shown to enhance AKT/ nuclear factor (erythroid-derived 2)-like 2 (Nrf2)/glutathione (GSH)-dependent antioxidant response in keratinocytes [11]. Moreover, this carotenoid reduces wrinkle formation and epidermal hypertrophy in mice [12] and suppresses melanogenesis and prostaglandin synthesis [13]. In addition, FX has been proposed as a photoprotective compound by stimulating restoration of the skin barrier in UVA-induced sunburn [14]. However, the protective effect of a FX-containing topical formulation has not been described yet on a skin inflammatory model. The possibility to administer FX topically has been subjected to several drawbacks because of its molecular weight and lipophilicity. As it is known, the compound must diffuse across *stratum corneum* and tight junctions to achieve effective permeation. In this sense, topical dosage forms such as creams, ointments, lotions and gels are commonly used for enhancing the cutaneous penetration. Thus, their composition will affect the drug permeation [15]. Concerning FX, several preparations have been studied with this aim [16,17]; in all of them, the evaluation of cutaneous penetration is a useful approach to predict the safety and efficacy of formulations [18]. To date, previous papers have developed topical experiments in mice applying acetone-dissolved compounds on skin [12,19]. With the aim of solving the irritant effect of acetone for its use in humans, a previous study from our group recently evaluated a glycolipid fraction-containing cream formulation on the murine 12-O-tetradecanoylphorbol 13-acetate (TPA)-induced epidermal hyperplasia model. Our findings reported an anti-inflammatory effect of this formulation with no signs of toxicity [20].

In the present study, we evaluated the anti-inflammatory and antioxidant properties of FX-containing cream in both in vitro and in vivo models in order to justify its use as adjuvant in inflammatory skin pathologies in which sun exposition is recommended. Firstly, we carried out two in vitro models to elucidate its anti-inflammatory and antioxidant activity. Then, FX was dissolved in absolute ethanol to further be incorporated into several common topical formulations (ointment, cream or hydrophilic gel). Once a topical formulation was selected, we finally aimed to study its effect on the TPA-induced hyperplasia model in mice, which mimics psoriatic parameters in dorsal murine skin, and on the UVB-induced erythema model in hairless mice, which reproduces the consequences expected in humans receiving acute UVB radiation.

## 2. Results

### 2.1. Effect of FX on Cell Viability

The effect of FX on THP-1 macrophages and HaCaT cells viability was measured by using the sulforhodamine B (SRB) assay. Results from cytotoxicity study showed that none of the tested concentrations affected cell viability. The inhibitory concentration 50 (IC_50_) (half maximal inhibitory concentration) was above 100 µM at 24 h after treatment (Table S1).

### 2.2. Effects of FX on TNF-α Production in LPS-Stimulated THP-1 Macrophages and IL-6 and IL-8 Production in TNF-α-Stimulated HaCaT Human Keratinocytes

**Figure 1:**
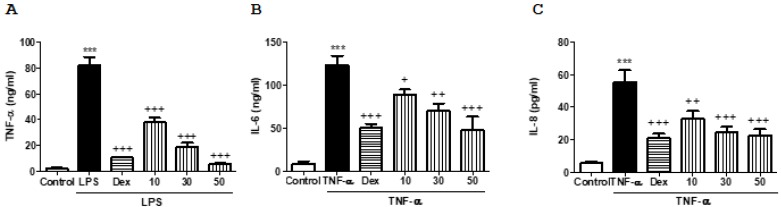
Effects of fucoxanthin (FX) on pro-inflammatory cytokines production in THP-1 macrophages and HaCaT keratinocytes. (**A**) Tumor necrosis factor alpha (TNF-α) in lipopolysaccharide (LPS)-stimulated THP-1 macrophages. (**B**) Interleukin (IL)-6 and (**C**) IL-8 in TNF-α-stimulated HaCaT keratinocytes. Cells were treated with FX (10, 30 and 50 μM) for 1 h, and then stimulated with LPS (1 μg/mL) in THP-1 macrophages or TNF-α (10 ng/mL) in HaCaT cells for 24 h. Dexamethasone (Dex) was used as positive reference compound at 1 μM. Production of cytokines in supernatant was measured by ELISA assay. Results are representative of six independent experiments. Values are means with standard errors represented by vertical bars. The mean value was significantly different compared with the control group (*** *p* < 0.001; Student *t* test). Mean value was significantly different compared with the LPS or TNF-α group (+ *p* < 0.05, ++ *p* < 0.01, +++ *p* < 0.001; one-way ANOVA followed by Bonferroni’s Multiple Comparison test).

Non-cytotoxic concentrations of FX were used to determinate its effect on pro-inflammatory cytokines in both THP-1 macrophages and human keratinocytes. As shown in Figure 1, all concentrations tested significantly reduced the production of inflammatory cytokines in both cell models. Interestingly, the pre-treatment with the carotenoid at 50 µM showed a significant reduction of tumor necrosis factor alpha (TNF-α) production in lipopolysaccharide (LPS)-stimulated THP-1 macrophages, reaching similar values to dexamethasone (Dex) (*p* < 0.001) (Figure 1A). In relation to interleukin (IL)-6 and IL-8 production in HaCaT keratinocytes, similar results were exhibited in cells pretreated with the highest dose of FX (*p* < 0.001) (Figure 1B,C).

### 2.3. In Vitro Permeation Studies of BC from Different Topical Formulations

**Figure 2:**
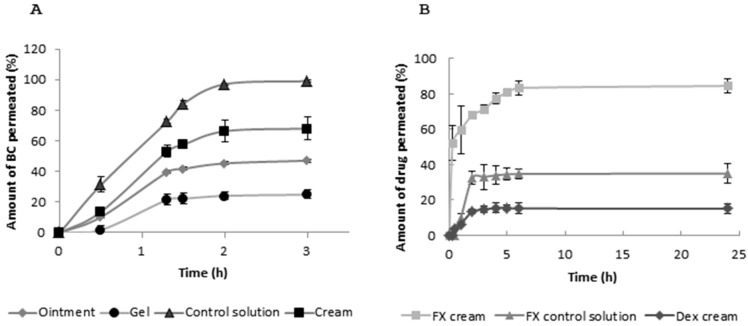
Permeation profiles. (**A**) In vitro permeation profile of β-Carotene (BC) from different topical formulations: cream, ointment and gel. BC dissolved in absolute ethanol was used as control solution. Artificial membranes impregnated with N-dodecanol mimicked the skin barrier. Absolute ethanol was used as release medium. (**B**) In vitro permeation profiles of fucoxanthin (FX) and dexamethasone (Dex) from cream formulation. FX dissolved in absolute ethanol was used as control solution. Dex-loaded cream was used as positive control. The experimental procedure was similar to that for BC. The percentages of drug release were obtained from the amount of drug that reached the receiver medium with time. These values are expressed in percentage, where 100% corresponds to the theoretical amount of drug added to the formulation. Data are represented as the mean ± standard error (*n* = 3).

The objective of this study was to evaluate the in vitro permeation through artificial membranes of β-carotene (BC) from different topical formulations. BC was selected because of its structural similarity with FX. The formulations tested were: ethanolic solution as control, hydrogel, cream and ointment. BC was detected in the receiver medium in a time-dependent manner and profiles of cumulative amount of drug permeated have been obtained (Figure 2).

The permeation profiles showed the cream as the most favourable vehicle as penetration enhancer. When compared with the ethanolic control solution, a high percentage of BC permeated was obtained at a flux very similar to the control solution, as reported in Table 1. However, the ointment and hydrogel preparations made it difficult the pass through the membrane, probably due to their lipophilic and hydrophilic nature, respectively. This was visualized in terms of lower permeation percentages and flux rates. Hydrophilic cream provided the highest release of BC in comparison with lipophilic ointment or hydrogels, as previously reported for flavonoids [21]. Therefore, the hydrophilic cream (oil-in-water (OW) emulsion base) was chosen for incorporating FX and Dex for further studies. As these emulsions have hydrophilic external phase, they are miscible with water and skin secretions, thus they are not occlusive and are easily removed from skin.

### 2.4. Ex Vivo Permeation Studies of FX-Containing Cream

Once the cream was selected as the vehicle for drug formulation, FX-loaded cream was prepared following the same methodology that for BC. In addition, Dex-loaded cream was formulated as positive control for the in vivo assay. The permeation profiles of FX contained in the hydrophilic cream were evaluated with the aim of analyzing the permeation behavior in the mice skin compared to an ethanolic control solution and Dex-cream.

Results showed a clear improvement of FX permeation with time (Figure 2). As evidenced from the area under the drug permeation curve (AUC) calculated (48,610, 19,914 and 8671%/h for FX-cream, FX control and Dex-cream, respectively), the cream vehicle offered a higher amount of FX permeated with time, whereas the control solution of this molecule showed a lower percentage. Although the ethanol acts as permeation enhancer, the cream composition, rich in surfactants, made the formulation improve the amount of FX to cross the membrane. Surprisingly, the permeation profile of Dex-cream showed 15%, approximately, of drug permeation. This lack of diffusion across the membrane could be attributed to a lower partition coefficient than FX (1.83 vs. 14.76). Dex has a lower molecular weight than FX, but the lipophilic nature of the carotenoid makes it a better candidate to interact with the surfactant components of the cream, which aids the solubilized molecule to reach the receiver medium.

### 2.5. Topical Application of FX-Containing Cream Decreases Skin Inflammation and Hyperplasia on the Murine TPA-Induced Model

**Figure 3:**
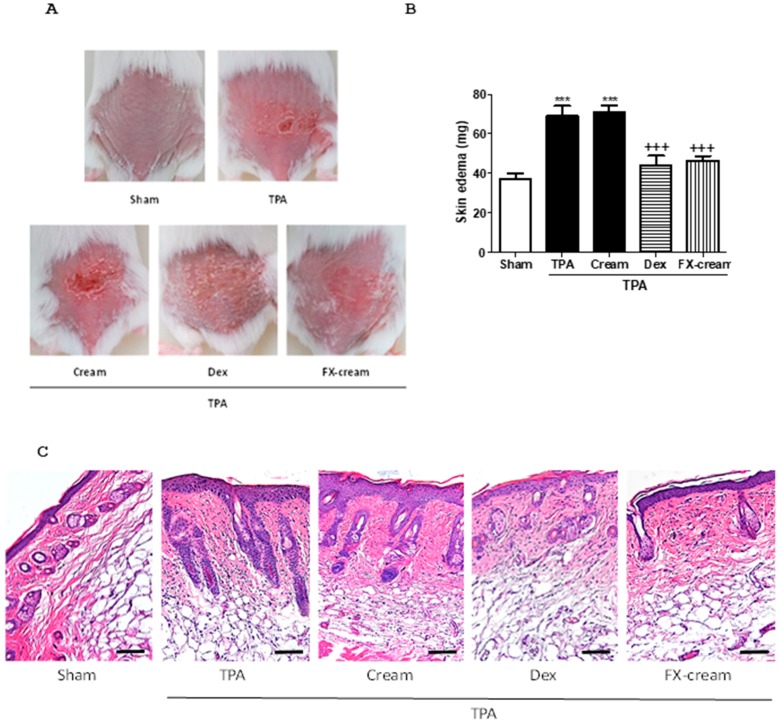

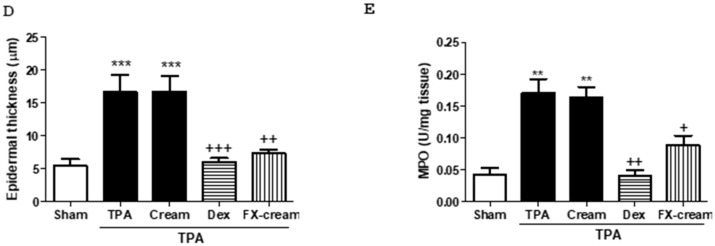
Fucoxanthin (FX) ameliorates skin hyperplasia and inflammation induced by 12-O-tetradecanoylphorbol-13-acetate (TPA) in mice (*n* = 10 mice/group). FX-cream formulation (100 mg per site at 200 µg), dexamethasone (Dex, 100 mg per site at 200 µg) or vehicle (cream with 0.2% ethanol) were topically administered as described in Material and methods. (**A**) Macroscopic mice back appearance at the end of experiment. (**B**) Skin edema as punch biopsy. (**C**) Histological appearance of mouse dorsal skin after hematoxylin/eosin (H&E)-staining; Bar = 10 mm. Original magnification 100× (**D**) Epidermal thickness assessment in H&E-stained skin slides. (**E**) Myeloperoxidase (MPO) activity. Values are means with standard errors represented by vertical bars. The mean value was significantly different compared with the sham group (** *p* < 0.01, *** *p* < 0.001; Student *t* test). The mean value was significantly different compared with TPA-cream group (+ *p* < 0.05, ++ *p* < 0.01, +++ *p* < 0.001; one-way ANOVA followed by Bonferroni’s Multiple Comparison test).

To analyze whether topical pre-treatment with FX could reduce the in vivo inflammation, we studied this carotenoid on the murine TPA-induced epidermal hyperplasia model. Topical pre-treatment with FX-cream (100 mg per site containing 200 µg of FX) and Dex (100 mg per site containing 200 µg of Dex) was administered from 2 days before TPA-induced hyperplasia and 1 h after each TPA application (2 nmol per zone for three consecutive days). Then, 24 h after the last application of TPA, mice were sacrificed and skin biopsies were removed and weighted. Mice treated with TPA or TPA-cream resulted in the development of macroscopic lesions as peeling (Figure 3A), confirmed by an increase of weight of the 1 cm^2^ punch biopsies of dorsal skin, in comparison with sham group (*p* < 0.001), with no significant differences between both groups (Figure 3B). This comparison led us to confirm that cream did not interfere in TPA action in our experiment. As expected, pre-treatment with the reference compound Dex significantly reduced the skin punch weight (*p* < 0.001). Similarly, mice treated with FX-cream showed a significant attenuation of skin edema when compared with TPA-cream group (*p* < 0.001) (Figure 3B). We next analyzed the skin homogenates to evaluate myeloperoxidase (MPO) activity as a neutrophil infiltration parameter with important relevance in hyperplasia model (Figure 3E). Our results confirm that this marker significantly increased after TPA application in comparison with sham group (*p* < 0.01). Dex application markedly diminished MPO activity in relation to TPA-cream group (*p* < 0.01). In a similar way, this parameter was reduced by FX-cream (*p* < 0.05) (Figure 3E). These results were confirmed with histological analysis of hematoxylin and eosin (H&E)-stained skin lesions in mice (Figure 3C), which showed that TPA administration produced a massive neutrophilic infiltration and an epidermal hyperplasia, because of uncontrolled and abnormal keratinocyte production. This effect was evidenced by a marked increase of epidermal thickness in this group (*p* < 0.001). Animals pre-treated with Dex and FX-cream evidenced a significant improvement in epidermal hyperplasia in relation to TPA-cream group (*p* < 0.001, and *p* < 0.01, respectively) (Figure 3C,D).

**Figure 4:**
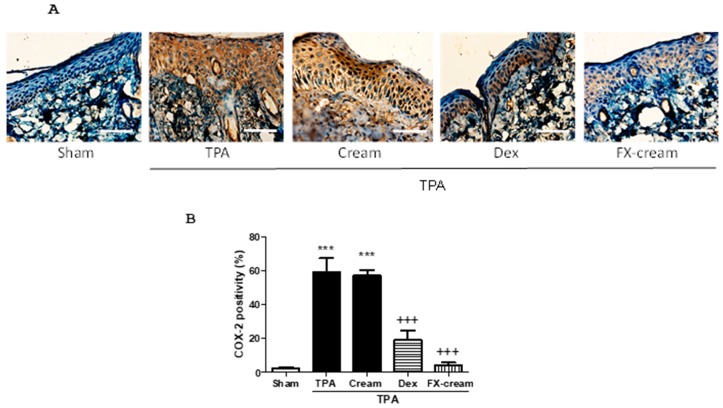
Topical application of fucoxanthin (FX) reduced 12-O-tetradecanoylphorbol-13-acetate (TPA)-induced epidermal cyclooxygenase-2 (COX-2) expression in mice. (**A**) Representative photographs of epidermal COX-2 distribution by immunohistochemical detection; Bar = 12.7 mm. Original magnification 200×. (**B**) Percentage of COX-2 positivity in epidermal layer was assessed by counting the number of COX-2 positive cells vs. total cells from 10 equal sections of immunostained dorsal skin per animal (*n* = 3). Values are means with standard errors represented by vertical bars. The mean value was significantly different compared with the sham group (*** *p* < 0.001; Student *t* test). The mean value was significantly different compared with TPA-cream group (+++ *p* < 0.001; one-way ANOVA followed by Bonferroni’s Multiple Comparison test).

To support the beneficial effects of FX on skin inflammation and explore its possible mechanism of action, we measured cyclooxygenase-2 (COX-2) expression in skin samples (Figure 4). This enzyme has been shown to have an important role in pathogenesis of skin diseases. Immunohistochemical analysis of this enzyme exhibited that TPA-induced hyperplasia significantly enhanced COX-2-positive cell numbers (*p* < 0.001), principally located in epidermal layer, in comparison with sham (Figure 4B). Figure 4 shows that the results of mice pre-treated with Dex confirmed the anti-inflammatory effect of this corticoid (*p* < 0.001). Interestingly, pre-treatment with FX-cream resulted in a marked decrease in the number of epidermal COX-2-positive stained cells related with TPA-cream group, reaching expression levels lower than those in Dex group (*p* < 0.001).

### 2.6. FX Protects Human HaCaT Keratinocytes against UVB-Caused Damage

**Figure 5:**
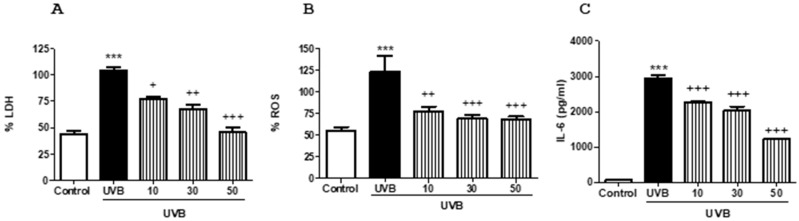
Protective effect of fucoxanthin (FX) on UVB-induced damage in HaCaT cells. Keratinocytes were preincubated with FX (10, 30 and 50 µM) for 1 h prior to UVB (50 mJ/cm^2^) exposure. (**A**) After 24 h, cell viability was assessed by using lactate dehydrogenase (LDH) assay. (**B**) Intracellular reactive oxygen species (ROS) generation was measured 30 min after UVB irradiation by relative fluorescence intensity using dichlorofluorescin-diacetate (DCF-DA) assay. (**C**) Interleukin (IL)-6 production was evaluated by ELISA assay, 24 after UVB exposure. Cell viability and ROS production are expressed as percentage respect to UVB-irradiated cells and IL-6 levels as pg/mL. Results are representative of four independent experiments. Values are means with standard errors represented by vertical bars. The mean value was significantly different compared with the control group (*** *p* < 0.001; Student *t* test). The mean value was significantly different compared with UVB group (+ *p* < 0.05, ++ *p* < 0.01, +++ *p* < 0.001; one-way ANOVA followed by Bonferroni’s Multiple Comparison test).

To examine the protective effect of FX in irradiated HaCaT cells, we evaluated cell viability by lactate dehydrogenase (LDH) enzyme activity, ROS levels and IL-6 production after UVB exposure. As expected, UVB irradiation significantly increased LDH activity, ROS content and IL-6 production in HaCaT keratinocytes compared to unirradiated control (*p* < 0.001) (Figure 5A–C). Pre-treatment of cells with FX 1 h prior to UVB exposure significantly decreased UVB-induced mortality, preserving cell membrane integrity in a dose-dependent manner at all concentrations used (10, 30 and 50 μM, *p* < 0.05, *p* < 0.01, *p* < 0.001, respectively) (Figure 5A). We next evaluated intracellular ROS levels in UVB-irradiated cells based on the dichlorofluorescein diacetate (DCF-DA) assay (Figure 5B). FX markedly reduced ROS levels by 23, 31 and 32% at the concentration of 10, 30 and 50 μM, respectively (*p* < 0.01, *p* < 0.001, *p* < 0.001) (Figure 5B).

It is known that UVB exposure induces abnormally augmented cytokine production from keratinocytes, leading to inflammatory skin disorders. To evaluate the effect of FX on IL-6 production, HaCaT cells were pre-treated for 1 h with this carotenoid, and then were exposed to UVB. Pre-treatment with FX at the concentrations tested of 10, 30 and 50 μM substantially inhibited this cytokine levels by 23, 31 and 59%, respectively (*p* < 0.001) (Figure 5C).

### 2.7. Topical Application of FX-Containing Cream Protects against UVB-Induced Skin Erythema in SKH-1 Hairless Mice

**Figure 6:**
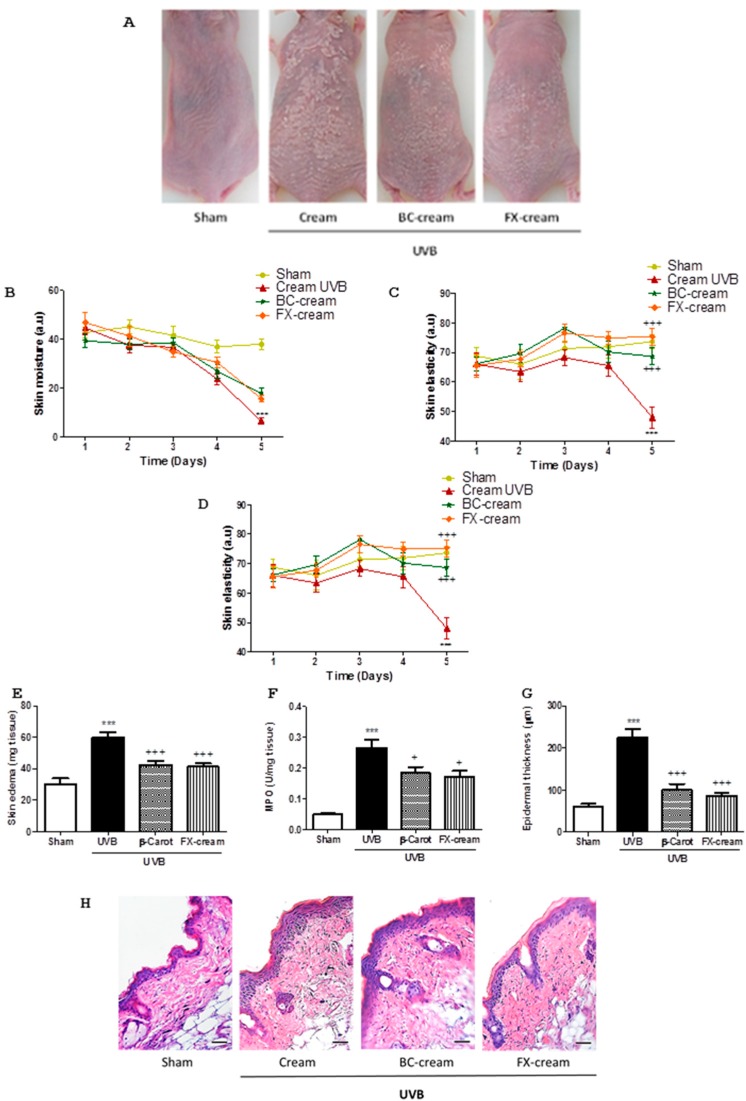
Fucoxanthin (FX) has photoprotective effects in UVB-induced erythema model in hairless mice (*n* = 8 mice/group). Mice received a single UVB radiation dose of 360 mJ/cm^2^. FX-cream formulation (FX-cream, 100 mg per site at 200 µg), β-carotene-cream (BC-cream, 100 mg per site at 200 µg) or vehicle (cream with 0.2% ethanol) were topically administered as described in Material and methods. (**A**) Macroscopic mice back appearance at the end of experiment. (**B**) Skin moisture, (**C**) skin elasticity and (**D**) melanin index were evaluated during all experiment. (a.u), arbitrary units. (**E**) Determination of skin edema as punch biopsy weight. (**F**) Myeloperoxidase (MPO) activity. (**G**) Measurement of the epidermal thickness in hematoxylin/eosin (H&E)-stained skin slides. (**H**) Photomicrographs of mouse dorsal skin after H&E-staining; Bar = 10 mm. Original magnification 100×. Values are means with standard errors represented by vertical bars. The mean value was significantly different compared with the sham group (*** *p* < 0.001; Student *t* test). The mean value was significantly different compared with UVB-exposed group (+ *p* < 0.05, +++ *p* < 0.001; one-way ANOVA followed by Bonferroni’s Multiple Comparison test).

The possible photoprotective effect of the topical pre-treatment with FX-cream in hairless mice was assayed using an acute proinflammatory UVB dose (360 mJ/cm^2^) [22]. BC-cream was employed as reference compound due to its antioxidant and photoprotective activity [23]. Animals were examined with a dermatoscope for five days, evaluating UVB-induced skin alterations. As shown in Figure 6A, a single acute UVB exposure accelerated skin damage, showing typical symptoms such as peeling, loss of moisture, reduction of elasticity and increase of melanin production in comparison with sham controls (Figure 6A–D). The progressive evaluation of mice showed that pre-treatment with FX-cream increased the skin moisture (Figure 6B) and elasticity (Figure 6C) and decreased the production of melanin (Figure 6D), with similar values to BC-cream. In order to confirm the protective profile of FX, 48 h after UVB irradiation, the mice were sacrificed and dorsal skin was examined. Our data reflected significant increases of skin edema and MPO activity in UVB-irradiated group when compared with sham (*p* < 0.001). The reference group, BC-cream, showed pronounced lower levels of these parameters when compared to UVB-irradiated group (*p* < 0.001, *p* < 0.05, respectively) (Figure 6E,F). Similar results were found after pre-treatment with FX-cream (Figure 6E,F). These findings were confirmed by histological study by using H&E staining. Histologically, a significant increase in superficial skin layer thickness due to unregulated keratinocytes proliferation was detected in UVB-irradiated animals in comparison to sham (*p* < 0.001) (Figure 6G,H). FX-cream treatment was as effective as topical application of BC-cream; our data revealed a decrease in keratinocytes proliferation when compared to UVB-irradiated group, evidenced by a significant reduction of epidermal thickness (*p* < 0.001) (Figure 6G,H).

**Figure 7:**
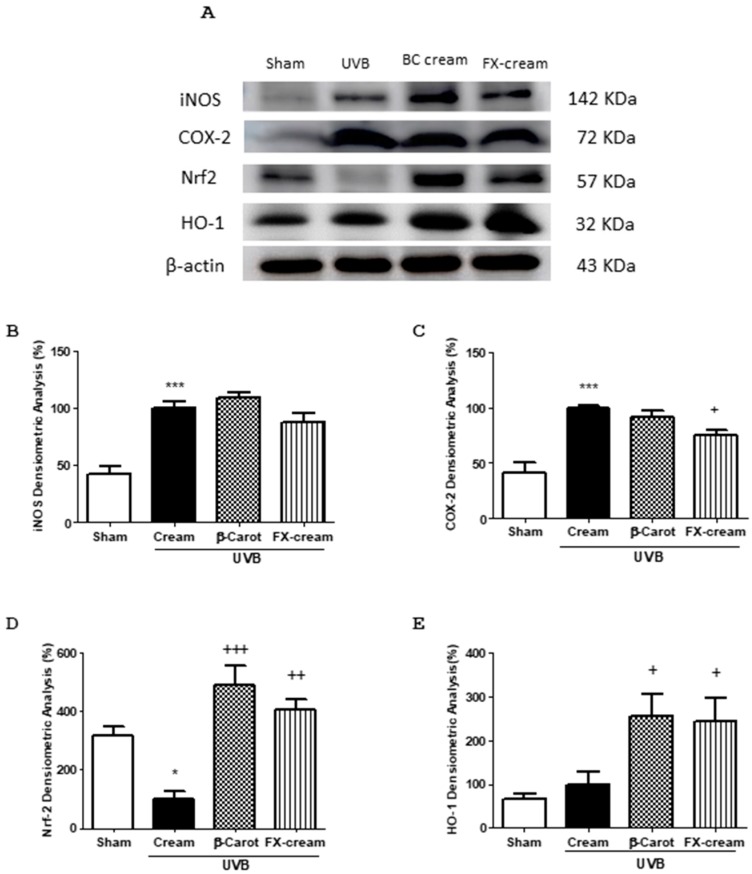
Anti-inflammatory and antioxidant effects of fucoxanthin (FX) in UVB-induced erythema model in hairless mice (*n* = 8 mice/group). Mice received a single UVB radiation dose of 360 mJ/cm^2^. FX-cream formulation (100 mg per site at 200 µg), β-carotene-cream (BC-cream, 100 mg per site at 200 µg) or vehicle (cream with 0.2% ethanol) were topically administered as described in Material and methods. (**A**) Representative Western blot images of different skin proteins. Densitometric analysis of (**B**) inducible nitric oxide synthase (iNOS), (**C**) cyclooxygenase-2 (COX-2), (**D**) nuclear factor (erythroid-derived 2)-like 2 (Nrf2) and (**E**) heme oxygenase-1 (HO-1) proteins. Data were studied following normalization to the control (housekeeping gene, β-actin). Values are means with standard errors represented by vertical bars. The mean value was significantly different compared with the sham group (* *p* < 0.05, *** *p* < 0.001; Student *t* test). The mean value was significantly different compared with UVB-exposed group (+ *p* < 0.05, ++ *p* < 0.01, +++ *p* < 0.001 one-way ANOVA followed by Bonferroni’s Multiple Comparison test).

To further explore the possible mechanisms of action of FX, we examined the expression levels of different inflammatory and antioxidant proteins in dorsal skin samples. Exposure of skin to UVB induced a significant increase in the pro-inflammatory enzymes iNOS (*p* < 0.001) and COX-2 (*p* < 0.001) expression (Figure 7B,C). Neither of these proteins changed significantly in animals treated with the reference compound BC-cream. However, pre-treatment with FX-cream resulted in a significant downregulation of COX-2 expression levels in irradiated mice (*p* < 0.05) in comparison with UVB-irradiated animals. As regards the iNOS protein, although its expression tended to decrease in FX-cream-treated animals, no significant differences were observed in relation to UVB-treated mice. UVB exposure significantly down-regulated the expression of Nrf-2 protein (*p* < 0.05). BC-cream application induced a marked increase in Nrf2 levels (*p* < 0.001) accompanied with a rise of its target gene heme oxygenase-1 (HO-1) (*p* < 0.05). In a similar way, FX-cream significantly increased the expression of these antioxidant proteins in UVB-exposed skin (*p* < 0.01 and *p* < 0.05, respectively) (Figure 7D,E).

## 3. Discussion

FX has formerly demonstrated in vitro and in vivo anti-inflammatory and antioxidant activities [11,14,24]. Nevertheless, the use of topical formulations such as cream/emulsion containing this carotenoid to prevent exacerbations related with skin inflammatory pathologies and provide a photoprotective effect has not been previously evidenced. Treatment of the skin with these topical formulations is a rational approach since avoids gastrointestinal degradation and preserves bioavailability [25].

Firstly, in the present study, we confirmed the in vitro anti-inflammatory activity of FX through decrease in TNF-α production in LPS-stimulated macrophages and reduction of IL-6 and IL-8 levels in TNF-α-stimulated HaCaT keratinocytes. These results led us to evaluate the possible preventive effects of this carotenoid on two in vivo acute skin inflammatory models: a hyperplasia model induced by multiple applications of TPA and an erythema model induced by a single UVB challenge.

Drug delivery across the skin remains as an important challenge in the development of drug delivery systems. This is mainly attributed to the highly organized intercellular lipids and poor permeability of *stratum corneum* [26]. It is also well-known that lipophilic molecules move across the barrier by a transcellular mechanism whereas hydrophilic agents are likely to follow a paracellular pathway to cross the skin. FX is lipophilic with a high partition coefficient (log P 6.83–7.54). To date, some studies with FX have been carried out by dissolving FX in ethanol for applying the drug dissolved onto the skin [12]. However, this solvent is known to produce dryness effect in the skin. To improve FX topical application, various hydrophilic and lipid-based formulations have been developed, firstly using BC as model drug because of its structural similarity to FX [27]. In this sense, new approaches are desirable in order to avoid the use of uncomfortable formulations such as paraffin [13]. In order to have a prediction of the in vivo permeation behaviour of the drug and to avoid the expensive cost of the use of animals or human skin, alternative artificial skin diffusion apparatus was used for conducting in vitro permeation studies. As regards the formulations tested (cream, ointment and hydrogel), it was found that lipid-based formulations improved most efficiently the diffusion of BC through the permeation membrane in a Franz diffusion cell. In contrast, water-based formulations, such as Carbopol gel, exhibited poor penetration. The composition of cream, which was highly charged in surfactants, makes it to act as a permeation enhancer that changes the atmosphere of the lipids to encourage the diffusion of lipid molecules (BC or FX) and/or influence their solubility [28]. The blend of agents with polar and non-polar properties, which probably mimic the complex lipid/polar nature of the *stratum corneum*, makes the cream thermodynamically similar to the *stratum corneum*, enabling the permeation of BC through the skin. Other authors incorporated surfactant-like molecules (D-α-tocopheryl polyethylene glycol 1000 succinate) into the formulations for synergistically acting with ethanol as solubilizing agents of griseofulvin [29]. This is the reason why formulations too occlusive and lipophilic (ointment) make difficult the permeation process of poorly water-soluble compounds. On the contrary, concerning the highly hydrophilic formulations (gel), it is known that water can generate a resistant boundary at the donor-skin interface (impregnated in our artificial membranes with dodecanol) and may prolong or delay the permeation of poorly water-soluble molecules such as BC.

Once we selected the cream as a vehicle for our studies, we proceeded to elucidate the FX behaviour in terms of permeation kinetics through a comparative study with control solution and Dex-cream. This carotenoid has been previously formulated in O/W formulations by other authors [16]. Our results showed that, under the experimental conditions used, FX-cream has a higher permeability though artificial membrane than the other formulations (control solution FX and Dex-cream). Other authors reported that the differences in penetration might be associated with the variation in the lipophilicity of the tested compounds [30], as obtained in this case. Although Dex has a lower molecular weight than FX, which might favour the diffusion across the membrane, its low lipophilicity (log P 1.83) compared to FX, makes more difficult this passage, showing permeation profiles more reduced [26]. The efficacy of FX-loaded O/W emulsions has been demonstrated as anti-obesity formulations being prepared by using medium chain triglyceride as an oil phase and l-α-phosphatidylcholine as an emulsifier [17,31]. This heterogeneous disperse system has been also used for incorporating other antioxidant molecules such as green tea polyphenols with the aim of preventing UVB-induced oxidation of lipids in mouse skin [21]. Thus, hydrophilic cream may serve as an optimal delivery system for FX use in animal models.

TPA is an activator of protein kinase C isoenzymes and a well-known inducer of inflammatory response in murine skin rising expression of the inflammation mediators [32]. TPA-induced hyperplasia model in dorsal murine skin reproduces typical manifestations of inflammatory skin pathologies as psoriasis. In this regards, TPA administration causes macroscopic lesions such as erythema or peeling, as well as increment of epidermis thickness by hyperproliferation and aberrant differentiation of keratinocytes or leukocytes infiltration into the dermis and consequent cytokines and chemokines production [33]. The pre-treatment with FX-cream ameliorated skin hyperplasia reducing MPO activity, as indicatory of leucocyte infiltration, and skin edema. These results were related with the improvement of histological damage by reducing epidermal thickness and neutrophil infiltration, getting similar levels to those of the reference corticosteroid Dex. The relation between epidermal differentiation and COX-2 expression has been strongly reported, as well as their connection with the pathogenesis of psoriasis [34]. In this sense, repeated applications of TPA in dorsal murine skin induce inflammation symptoms that are accompanied by COX-2 epidermal overexpression in relation with healthy group [35]. Our data evidenced a reduced COX-2 expression in mice treated with FX, presenting lower levels than those in Dex group. These findings, at least partly, suggest that suppression of COX-2 expression may be involved in the preventive effect of this carotenoid on TPA-induced epidermal hyperplasia. Our results are in agreement with previous in vitro studies that showed the capacity of FX to modulate inflammatory response through inhibition of COX-2 and iNOS expression and the consequent decrease of NO and prostaglandin E2 levels [24,36]. The molecular mechanisms underlying the anti-inflammatory properties of FX may be associated with the suppression of nuclear factor-kappaB and mitogen-activated protein kinase pathways, similar to those previously reported for Dex in skin inflammatory pathologies [37]. More recently, FX has shown to downregulate COX-2 levels in a murine model of high-fat-diet-induced obesity [38]. It is well known that inflammatory pathologies as psoriasis require long-term topical corticosteroids therapy, which is associated with both topical and systemic adverse effects. Moreover, the chronic use of these compounds may increase tendency to “steroid addiction syndrome”, which forces to change the corticoid and select one more potent [39]. For these reasons, the use of a well-tolerated immune-modulatory topical formulation containing FX could offer a safer alternative to continued use of corticoids for the treatment of skin inflammatory disorders.

Additionally, it is worth highlighting that since these pathologies benefit from therapeutic sun exposition, it is very important to assure the photoprotection of exposed skin. Sunscreens are used to prevent the deleterious effects of UVB due to this radiation remains as an important risk factor to develop skin lesions [40]. In this sense, further therapeutic strategies targeting UVB-induced inflammation and oxidative stress are necessary. Thus, our next objective was to examine the photoprotective effects of FX in an UVB-induced cell damage model in HaCaT keratinocytes, as well as on an UVB-induced erythema model in hairless mice in order to complete the functional activity study of this compound as adjuvant treatment in skin inflammatory disorders. It is well known that UVB irradiation induces cell cytotoxicity through loss of cellular membrane integrity, which leads to liberation of LDH enzyme from cytosol to the culture medium [41]. Moreover, UVB radiation causes increased ROS production and DNA damage, as well as a strong inflammatory response, characterized by the production of inflammatory cytokines, such as IL-6, which leads to premature skin aging and carcinomas [42]. Recently, it has been demonstrated that the antioxidative role of FX in HaCaT keratinocytes is related to DNA protection against oxidative stress and the prevention of apoptosis [43,44]. According with these results, we have shown that pre-treatment of HaCaT keratinocytes with FX significantly attenuated UVB-induced damage by increasing cell viability and inhibiting ROS and IL-6 levels.

In relation to in vivo alterations, acute UVB radiation promotes erythema, edema and loss of skin moisture and elasticity, as well as increases melanin production, which is involved in melanogenesis. Recently, several studies have revealed the photoprotective effects of FX in UVB-irradiated mice by reducing melanogenesis parameters [13] and in UVA-induced sunburn by promotion of skin barrier formation [14]. In addition, this carotenoid prevented skin photoaging in hairless mice through its antiangiogenic and antioxidant effects [12]. Herein, we report that the topical pre-treatment with FX-cream formulation ameliorated erythema induced by UVB as well as edema and MPO activity, which are important inflammation and neutrophil recruitment parameters. Overexpression of COX-2 is highly related to some carcinomas including skin cancer [45]. In this regard, and in accordance to the above results from TPA model, we also reported the reduction of COX-2 expression by FX-cream pre-treatment on UVB-exposed skin, effect not observed upon BC administration.

It has been reported that excessive ROS generation results in oxidative stress in skin cells and plays a vital role in the initiation, promotion and progression of skin aging, carcinogenesis and many inflammatory disorders [46]. In this respect, Nrf2 is a transcription factor that perceives variation in cellular oxidative stress and promotes the transcription of antioxidant genes and detoxification enzymes such as HO-1 to protect against UVB-induced oxidative damage [47,48]. An in vitro study has elicited the antioxidant activity of FX by enhancing the Akt/Nrf2/GSH pathways in human keratinocytes [11]. Moreover, FX has been recently shown to be able to activate Nrf2 signalling pathway by inducing demethylation of CpG sites in Nrf2 [49]. In accordance with these results, our data reported that FX and BC offer a similar protection against oxidative stress caused by UVB exposure via increase of Nrf2 expression and its downstream target HO-1. However, in a previous paper FX was confirmed to have higher antioxidative effects than BC, due to its more polar nature and consequent placement in the cell membrane [50]. In the present study, although the two carotenoids have shown to have similar antioxidant properties, FX provides additional benefits, exhibiting an anti-inflammatory activity by downregulating COX-2 expression after UVB irradiation. These findings propose this carotenoid as a natural approach for protecting skin against UV radiation and modulating the inflammatory response associated.

## 4. Materials and Methods

### 4.1. Cell Culture

The THP-1 human monocytic leukemia cell line and HaCaT human keratinocytes were obtained from the American Type Culture Collection (ATCC, Manassas, VA, USA). THP-1 cells were cultured in RPMI 1640 medium (GIBCO, Grand Island, New York, NY, USA) containing 10% heat-inactivated fetal bovine serum (FBS), 100 U/mL penicillin and 100 μg/mL streptomycin. HaCaT human keratinocytes were maintained in high glucose Dulbecco’s modified Eagle’s medium (DMEM, GIBCO, Grand Island, New York, NY, USA). Both cell lines were grown in a humidified atmosphere containing 5% CO_2_ at 37 °C [20].

### 4.2. Cell Viability Assay

SRB assay was used for determining the viability of THP-1 macrophages and HaCaT cells upon exposure to FX (Sigma-Aldrich, St. Louis, MO, USA) [51]. Firstly, for differentiation into macrophages, THP-1 cells were seeded into 96-well plates at 10^4^ cells/well in presence of phorbol 12-myristate 13-acetate (PMA, Sigma-Aldrich, St. Louis, MO, USA) for a final concentration of 0.2 µM for 72 h in 96-well plates and HaCaT cells were seeded into 96-well plates in the growth medium at 10^4^ cells/well for 24 h to ensure the adherence. Both cellular types were incubated in a humidified atmosphere of 5% CO_2_ at 37 °C. After that, cells were treated with FX at final concentrations range of 10–100 µM in DMSO 0.1% (*v*/*v*) and the cytotoxicity was measured after 24, 48 and 72 h of incubation. The absorbance was determined at 492 nm in a microplate spectrophotometer (Sinergy HT, Biotek^®^, Bad Friedrichshall, Germany).

### 4.3. Determination of TNF-α Production

THP-1 monocytes were differentiated into macrophages in 96-well plates (10^4^ cells/well). Then, cells were incubated for 1 h with FX (10, 30 and 50 µM). The positive reference compound used was Dex (1 µM) (Sigma-Aldrich, St. Louis, MO, USA). Inflammatory response was induced by addition of LPS (1 μg/mL) except for control group [52]. After 24 h, commercial enzyme-linked immunosorbent assay (ELISA) kit (Diaclone GEN-PROBE, Besançon, France) was used to quantify TNF-α according to the manufacturer’s protocol.

### 4.4. Determination of IL-6 and IL-8 Production

HaCaT cells were seeded in 6-well plates (5 × 10^5^ cells/well). After 24 h, cells were washed twice (PBS, 4 °C) and medium containing FX (10, 30 and 50 μM) or Dex (1 μM) was added for 1 h, and then cells were stimulated with TNF-α (10 ng/mL) except for control group. After 24 h, supernatant fluids were collected and stored −80 °C until measurements [20]. Commercial enzyme-linked immunosorbent assay (ELISA) kit (Diaclone GEN-PROBE, Besançon, France) was used to quantify IL-6 and IL-8 according to the manufacturer’s protocol.

### 4.5. Preparation of Topical Formulations

Based on previous studies, topical formulations were performed following the methodology detailed in Rodríguez-Luna et al. [20]. Thus, three different preparations were developed: hydrogel, cream and ointment. Concerning the hydrogel, BC (Sigma-Aldrich, St. Louis, MO, USA) solution in ethanol absolute (10 mg/mL) was gradually added to the polymer dispersion (1% *w*/*v*) under magnetic stirring. The ointment was prepared using both the melt emulsification and stirring steps. BC dissolved in ethanol (10 mg/mL) was added to the lipid mixture. Regarding cream, three different preparations were made containing 2 mg/g of drug (BC, FX or Dex). In all of them, the drug was dissolved in absolute ethanol (10 mg/mL) and added to the excipient cold mixture.

### 4.6. In Vitro Permeation Studies from Topical Formulations

In vitro drug permeation studies from the topical formulations were performed using a Franz diffusion cell apparatus (SES-Gmgh Analyses system, Bechenheim, Germany), with 14.5 mL receptor volume and 3.14 cm^2^ diffusion area. An appropriately conditioned cellulose nitrate membrane (Tuffryn^®^; Pall Corporation, Port Washington, New York, NY, USA), impregnated with lauryl alcohol (membrane weight increase 90–110%), was employed as artificial lipophilic membrane simulating the epidermal barrier [53,54]. The membrane was placed in the diffusion cell sandwiching the donor and receptor compartments. The receiver solution contained absolute ethanol and the donor chamber was filled with 1 g of formulation at 2 mg/g of BC (cream, ointment or hydrogel). In this study, BC was employed as reference compound because of its structural similarity with FX. The whole diffusion cell was thermostated maintaining the temperature at 32 °C. Aliquots of 0.5 mL were withdrawn from the receptor chamber after 0.5, 1, 1.5, 2 and 3 h and replaced with fresh ethanol. The concentrations of BC were spectrometrically assayed at 454 nm (UV/vis 1601 Shimadzu, Duisburg, Germany). The cumulative amounts of permeated drug per unit area in the receiver chamber (µg/cm^2^) were plotted as a function of time (h). The slope of the linear portion of the plot was presented as steady state flux (Jss, µg/cm^2^/h). From the permeation profiles, the AUC was calculated using the trapezoidal rule as a quantitative parameter for studying the permeation magnitude [55].

### 4.7. Animals

Female Swiss CD-1 mice (25–30 g) were purchased from Janvier-Labs (Le Genest St Isle, France) and female SKH-1 hairless mice (18–20 g) from Charles River Laboratories (Écully, France). Mice were maintained under standard conditions (temperature of 24–25 °C, humidity of 70–75% and 12 h light-12 h dark cycle) and were allowed free access to a standard diet (Panlab) and water *ad libitum*. All studies were performed in accordance with the recommendations of the European Union regarding animal experimentation (Directive of the European Council 2010/63/EU). The experiments followed protocols approved by the Animal Ethics Committee of the University of Seville (Protocol 06/04/2018/042).

### 4.8. Ex Vivo Permeation Studies from Creams

Once the cream carrier was chosen from the previous study, ex vivo diffusion studies were performed using excised mice skin as a membrane for evaluating the permeation behaviour of FX-loaded cream. Moreover, Dex-cream was included because it was used as positive control in the in vivo hyperplasia study. Swiss CD-1 mice were sacrificed by cervical dislocation and full dorsal skin was excised. A specific portion of the skin was washed with distilled water and cut for the permeation assay. The study was carried out following the methodology previously reported [56]. Animal skin was inserted between the donor and receiving compartments and adjusted by means of a pinch clamp. The receiving chamber was filled with 14.5 mL of degassed ethanol absolute and was thermostated by means of a water bath circulator and a jacket surrounding the cell, maintaining 32 °C in the skin surface. The receiving medium was continuously stirred to avoid diffusion layer effect.

FX-cream (0.2% *w*/*w*), FX control solution in ethanol (0.2% *w*/*w*) and Dex-cream (0.2% *w*/*w*) were accurately measured and placed on stratum corneum in the donor compartment and sealed with parafilm. The same procedure as previously described was followed [56]. Absolute ethanol was used as solvent in the receiver compartment. The concentrations of Dex were spectrometrically assayed at 254 nm (UV/vis 1601 Shimadzu). The FX content in the ethanol solutions was analyzed by using HPLC method. The analysis was performed on a Hitachi Elite Lachron HPLC system equipped with a L-2130 isocratic pump, a diode array detector L-2455 and a L-2200 autosampler. Separation was carried out within a chromatographic C18 column (Merck, RP-18 LichroCART^®^ 150 mm × 4 mm, 5 µm). For drug analysis, the injection volume was 10 µL, and the flow rate and column temperature were set at 1.0 mL/min and 25 °C, respectively. The mobile phase consisted of A (formic acid 0.1%) and B (acetonitrile). The elution program was: 6 min isocratic at 90% B, followed by a gradient to 100% B at 10 min. Afterwards, the column was re-equilibrated during 5 min at 90% B. The cumulative amount of drug in receptor chamber for the three formulations (FX-cream, FX control solution and Dex-cream) was measured as a function of time (t, h). The cumulative amount (%) of drug permeated through the skin (*P*%) was determined as per the following equation [57]:

(1) $$P\%=\frac{C_{n}\cdot V+\sum_{i=1}^{n-1}C_{i}\cdot V_{i}}{M}\cdot 100$$

where *C_n_* is the drug concentration of the *n*th sampling point (mg/mL), *C_i_* is the drug concentration of the *i*th sample point (mg/mL), *V* is the total volume (14.5 mL) of liquid in receiving pool, *V_i_* is the volume (0.5 mL) of the *i*th sampling points and *M* is the mass of drug (FX or Dex) [20].

### 4.9. TPA-Induced Epidermal Hyperplasia Model and Treatments

Female Swiss CD-1 mice (*n* = 10 per group) were used to study the effect of FX on TPA-induced hyperplasia in mice dorsal skin. Briefly, the dorsal hair of animals was removed with an electric clipper and treated with depilatory cream (Deliplus, Barcelona, Spain) [35]. After 24 h, the animals were assigned to the different groups: Sham (vehicle: 100 mg of cream with 0.2% of ethanol); TPA control group; TPA-Cream (vehicle: 100 mg of cream with 0.2% of ethanol); Dex-cream (100 mg of cream per site, containing 200 µg of Dex dissolved in ethanol at 10 µg/mL); FX-cream formulation (100 mg of cream per site, containing 200 µg of FX dissolved in ethanol at 10 µg/mL). The creams were applied to the dorsal skin in an area of 1 cm^2^ using a syringe, during 2 days before the hyperplasia induction. Mice were anesthetized with ketamine (100 mg/kg of animal) and diazepam (5 mg/kg of animal) during treatments and TPA challenge. On day 4, TPA (2 nmol of TPA, dissolved in 20 µL of ethanol) was topically applied to the same areas on all groups except in sham group. After 1 h, FX-cream, the Dex-cream or vehicle were administered following the mentioned protocol. This procedure was replicated during two consecutive days. After 24 h of the last TPA dorsal application (day 7), mice were sacrificed by cervical dislocation and punch biopsies from the treated dorsal skin were weighed to evaluate edema, before further processing for histology and biochemical parameters.

### 4.10. MPO Activity

The measurement of MPO activity was used as a marker of neutrophil infiltration [58]. The tissue was thawed, weighed and homogenized in 10 volumes of 50 mM-PBS (pH 7.4). Then, the homogenate was centrifuged at 20,000× *g* for 20 min at 4 °C and the pellet was again homogenized in 10 volumes of 50 mM-PBS (pH 6) containing hexadecyl trimethylammonium bromide (0.5%) and 10 mM-EDTA. This homogenate was subjected to one cycle of freezing/thawing and sonicated. The homogenate samples (50 µL) were added to 96-well microplate and incubated at 37 °C for 3 min with a measurement mix (*o*-dianisidine dihydrochloride (0.067%), hexadecyl trimethyl-ammonium bromide (0.5%) and 0.3 mM-H_2_O_2_). The changes in absorbance were monitorized at 450 nm with a microplate reader (Labysystem Multiskan EX, Thermo Scientific, New York, NY, USA). MPO activity unit was defined as the amount of enzyme present that produced a change in absorbance of 1.0 unit/min at 37 °C in the final reaction volume containing acetate. Results are expressed as units/mg tissue [20].

### 4.11. Histological Study

The samples were fixed in paraformaldehyde, dehydrated by increasing concentrations of ethanol and embedded in paraffin. For H&E stains, tissue sections were cut to 7 μm on a rotary microtome (Leica Microsystems, Wetzlar, Germany), mounted on slides, deparaffinized with xylene, rehydrated through graded alcohols and stained according to standard protocols. All tissue sections were examined under an Olympus BH-2 microscope (GMI Inc., Ramsey, MN, USA) for determination of histopathological changes. Epidermal thickness was measured by using Scientific Imaging Systems (Biophotonics ImageJ Analysis Software; National Institutes of Health, Rockville, MD, USA).

### 4.12. Immunohistochemical Analysis

COX-2 expression was measured by immunohistochemical analysis by using a streptavidin-biotin-peroxidase method [59]. Paraffin-embedded dorsal skin sections (7 µm) were mounted on slides, deparaffinized with xylene and rehydrated through graded alcohols. These sections were boiled (10 mM citrate buffer (pH 6.0) for 3 min) for antigen retrieval, followed by cooling at room temperature for 20 min. Endogenous peroxidase was quenched with 0.3% (*v*/*v*) hydrogen peroxide for 20 min and the sections were washed (PBS, 10 min). To minimize the non-specific adsorption the sections were incubated in normal horse serum (Vectastain Kit; Vector Laboratories, Burlingame, CA, USA) for 20 min. Afterwards, slides were incubated with rabbit polyclonal anti-COX-2 antibody (Cayman Chemical, Ann Arbor, MI, USA) (1:300) overnight at 4 °C. Then, the samples were treated with anti-mouse IgG antibody. After 30 min, the cells were incubated with the streptavidin–peroxidase complex for 30 min, at room temperature (Vectastain Kit; Vector Laboratories, Burlingame, CA, USA). The enzymatic activities were developed with 3,3′-diaminobenzidine (DAB), and the sections were counterstained with hematoxylin. Negative control sections were treated in the same way, omitting the primary antibody. To examine COX-2 immunoreactivity, the microscope Olympus BX61 was used (Olympus Optical Co. Ltd., Tokyo, Japan). The quantification of immunohistochemical data was done by counting the number of immunostained cells as percent of total epidermal cells from 10 microscopic fields of immunostained tissues per animal.

### 4.13. UVB Irradiation of HaCaT Keratinocytes

Human keratinocytes were exposed to UVB radiation as previously described [60]. Briefly, the cells were grown to confluence and were treated with different concentrations of FX (10, 30 and 50 µM) for 1 h. Then, the medium was removed and a thin layer of PBS was added. Cells were exposed to a single dose of UVB radiation (50 mJ/cm^2^) for 1 min. The UVB source was a CL-1000M UV Crosslinker (UVP, Upland, CA, USA), which was used to deliver an energy spectrum of UVB light (280–315 nm; peak intensity, 302 nm). After UVB irradiation, the cells were supplied with fresh complete medium and incubated for 24 h.

### 4.14. Analysis of Intracellular LDH Activity

The measure of cytosolic enzyme LDH is one of the commonly used methods for assessing loss of cellular membrane integrity. The assay is based on the conversion of lactate to pyruvate in the presence of LDH with the consequence oxidation of NADH as previously described [61]. HaCaT cells were seeded in 6-well plates (5 × 10^5^ cells/well). After 24 h, cells were treated with FX (10, 30 and 50 μM) for 1 h and then, were irradiated with UVB. After 24 h, cell-free supernatants and cell lysates were mixed in a 96-well plate and the absorbance was read by using a microplate reader system (Sinergy HT, Biotek^®^, Bad Friedrichshall, Germany). LDH leakage was estimated calculating the LDH activity in the cell-free medium and LDH activity in lysates ratio. Results were represented as the percentage (%) of change in activity compared with the control cells.

### 4.15. Intracellular ROS Scavenging Activity

For quantification of ROS in HaCaT cells, the DCF-DA assay was employed [62]. HaCaT cells were seeded in nin96-well plates (10^4^ cells/well). Non-irradiated cells were used as negative control. After 24 h, cells were treated with FX (10, 30 and 50 µM) for 1 h and then, were exposed to UVB. Post irradiation, cells were incubated with 2′,7′-dichlorodihydrofluorescein diacetate (DCFH-DA) solution (5 mg/mL) in PBS for 30 min [63]. The fluorescence of the 2′,7′-dichlorofluorescein (DCF) product was determined using a fluorescence plate reader (Sinergy HT, Biotek^®^, Bad Friedrichshall, Germany) at 485 nm for excitation and 535 nm for emission.

### 4.16. Determination of IL-6 Production in UVB-Exposed HaCaT Keratinocytes

HaCaT cells (5 × 10^5^ cells/well) were seeded in 6-well plates. After 24 h, cells were treated with FX (10, 20 and 50 μM) for 1 h and then, were exposed to UVB. After 24 h, supernatant fluids were collected and ELISA kit (Diaclone GEN-PROBE, Besançon, France) was employed to quantify IL-6 according to the manufacturer’s protocol. The absorbance at 450 nm was read by a microplate reader (Labysystem Multiskan EX, Thermo Scientific, New York, NY, USA).

### 4.17. UVB-Induced Erythema in Hairless Mice

Female SKH-1 hairless mice (*n* = 8 per group) were used to study the effect of FX on UVB-induced erythema. Animals were distributed to the different groups: Sham (vehicle: 100 mg of cream with 0.2% of ethanol); UVB (100 mg of cream with 0.2% of ethanol); BC-cream (100 mg of cream per site, containing 200 µg of BC dissolved in ethanol at 10 µg/µL) as reference control; FX-cream (100 mg per site, containing 200 µg of FX dissolved in ethanol at 10 µg/µL). The formulations were applied on the dorsal skin (1 cm^2^/area) by using a syringe, and starting 2 days before irradiation. On day 3, 30 min after application of the substances, all groups except the sham control were exposed to an acute UVB dose (360 mJ/cm^2^) as previously described [22]. Then, 48 h after UVB exposure, mice were macroscopically evaluated and sacrificed. At this end-point time, a pronounced UVB-induced cutaneous inflammation was shown [4].

### 4.18. Dermatoscope Measurements

Dorsal skin was macroscopically examined every day by using a multi-dermatoscope (Dermatoscope MDS 800, Microcaya, Bilbao, Spain). Corneometer^®^ was used to determinate the hydration in the *stratum corneum* through electrical capacitance of the skin surface [64]. A Cutometer^®^ probe was used as suction method to analyse the real elasticity of skin [65]. In the last step, Mexameter^®^ was employed to analyse the redness skin by melanin index [66]. Every day, dorsal skin of mice was assessed twice and finally the results were expressed in arbitrary units (a. u.) or by a melanin index scale (1-36).

### 4.19. Western Blot Assay

Frozen dorsal skin tissues from UVB-induced erythema model were randomly selected (6 per group), weighed and homogenized in ice-cold buffer (50 mM Tris-HCl, pH 7.5, 8 mM MgCl_2_, 5 mM ethylene glycol bis (2-aminoethyl ether)-N,N,N′,N′-tetra acetic acid, 0.5 mM EDTA, 0.01 mg/mL leupeptin, 0.01 mg/mL pepstatin, 0.01 mg/mL aprotinin, 1 mM phenylmethylsulfonyl fluoride, and 250 mM NaCl). The homogenates were centrifuged (12,000× *g*, 15 min, 4 °C), and the supernatants were collected and stored at −80 °C. To determinate the protein concentration of the homogenates was used the Bradford colorimetric method [67]. Samples of the supernatants with equal amounts of protein (25 μg) were separated on 10% acrylamide gel by sodium dodecyl sulfate polyacrylamide gel electrophoresis. Then, the proteins were electrophoretically transferred onto a nitrocellulose membrane and incubated with specific primary antibodies: rabbit anti-inducible nitric oxide synthase (iNOS) (1:1000; Stressgen-Enzo Life Sciences, Farmingdale, NY, USA); rabbit anti-COX-2 (1:3000; Cayman Chemical^®^, Ann Arbor, MI, USA); rabbit anti-Nrf2, rabbit anti-HO-1 (1:1000; Cell Signaling, Danvers, MA, USA) overnight at 4 °C. After rinsing, the membranes were incubated with the horseradish peroxidase-linked (HRP) secondary antibody anti-rabbit (1:1000; Cayman Chemical^®^, Ann Arbor, MI, USA) or anti-mouse (1:1000; Dako^®^, Atlanta, GA, USA) containing blocking solution for 1 h at room temperature. To prove equal loading, the blots were analyzed for β-actin expression using an anti-β-actin antibody (1:1000; Sigma Aldrich^®^, St. Louis, MO, USA). Immunodetection was performed using an enhanced chemiluminescence light-detecting kit (SuperSignal West Pico Chemiluminescent Substrate, Pierce, IL, USA). Then, the immunosignals were monitorized by using LAS-3000 Imaging System (Fujifilm Image Reader, Valhalla, NY, USA) and densitometric data were studied after normalization to the control (housekeeping gene). The signals were analyzed and quantified with a Scientific Imaging Systems (Biophotonics ImageJ Analysis Software, National Institute of Mental Health, Bethesda, MD, USA) and expressed as percentage respect to sham control group [68].

### 4.20. Statistical Analysis

All values in the figures and text are expressed as arithmetic means ± SEM. Data were evaluated with GraphPad Prism version 5.00 software (GraphPad Software, Inc., San Diego, CA, USA). In all cases, the Shapiro-Wilk test was used to verify the normality of the data. The Mann-Whitney U-test was chosen for non-parametric values. The parametric values groups were analyzed by one-way analysis of variance (ANOVA) followed by Bonferroni’s Multiple Comparison Test. *p* values < 0.05 were considered statistically significant.

## 5. Conclusions

In conclusion, the in vitro anti-inflammatory, antioxidant and protective activity of FX has been confirmed in human keratinocytes. Furthermore, we have demonstrated for the first time the anti-inflammatory activity of a topical formulation containing FX in hyperplasic skin, reducing cell infiltrate and epidermal COX-2 expression, which has a main role in the progression of hyperplasia and the consequent skin damage. In addition, this carotenoid protects mice against superficial skin damage induced by UVB through inhibition of inflammatory mediators and promotion of antioxidant responses through Nrf2 pathway. For these reasons, FX, administered in a well-tolerated topical formulation and that improves its permeation, could be a novel natural adjuvant for preventing exacerbations associated with skin inflammatory pathologies as well as protecting skin against UV radiation.

## Figures and Tables

**Table 1:** Total percentage permeated across artificial lipophilic membrane of β-carotene (BC) from different topical formulations, and flux rate (Jss) (µg/cm^2^·min) calculated from the slope of amount permeated per area versus time.

Formulation	% Permeation (180 min)	Flux (Jss) (μg/cm^2^·min)	r^2^
Cream	68.38	0.1159	0.9795
Ointment	47.31	0.0548	0.9704
Hydrogel	25	0.0383	0.9403
Control	100	0.1350	0.9862

## Supplementary Materials

The following is available online at http://www.mdpi.com/1660-3397/16/10/378/s1, Table S1: Viability of THP-1 human macrophages and HaCaT human keratinocytes cells treated with different concentrations of fucoxanthin (FX). Values are mean ± ES (%) of three independent experiments (*n* = 3).

## Abbreviations

BC	β-carotene
COX-2	cyclooxygenase-2
DCF	2′,7′-dichlorofluorescein
DCF-DA	dichlorofluorescein diacetate
DCFH-DA	2′,7′-dichlorodihydrofluorescein diacetate
Dex	dexamethasone
FX	fucoxanthin
H&E	hematoxylin and eosin
HO-1	heme oxygenase-1
IL	interleukin
LDH	lactate dehydrogenase
LPS	lipopolysaccharide
MPO	myeloperoxidase
Nrf2	nuclear factor E2-related factor 2
ROS	reactive oxygen species
SRB	sulforhodamine B
TNF-α	tumor necrosis factor alpha
TPA	12-O-tetradecanoylphorbol-13-acetate
UV	ultraviolet
