# Supplementary material for: Fucoxanthin-Containing Cream Prevents Epidermal Hyperplasia and UVB-Induced Skin Erythema in Mice
Source: Mar Drugs. 2018 Oct 10;16(10):378. doi: 10.3390/md16100378 (PMC6212948; doi:10.3390/md16100378)
Supplement: Supplementary file 1 [file marinedrugs-16-00378-s001.pdf]

Supplementary Table S1. Viability of THP-1 human macrophages and HaCaT human keratinocytes cells treated with different concentrations of fucoxanthin (FX). Values are mean ± ES (%) of three independent experiments (*n* = 3).

	% Viability THP-1 macrophages (24 h)				
(μM)	10	30	50	100	IC_{50}
FX	97.5 ± 2.0	96.5 ± 1.2	103.7 ± 1.1	90.7 ± 0.5	> 100

	% Viability HaCaT keratinocytes (24 h)				
(μM)	10	30	50	100	IC_{50}
FX	111.2 ± 2.1	105.2 ± 4.0	103.9 ± 3.2	101.5 ± 2.2	> 100
